# Clinical Study of Paliperidone Palmitate Long-Acting Injection Combined With Electroacupuncture in the Treatment of Methamphetamine Addicts

**DOI:** 10.3389/fphar.2021.698740

**Published:** 2021-06-17

**Authors:** Yu Chen, Mingchao Li, Qiuming Ji, Zou Su, Ziyu Yang, Yin Xu, Qian Chen, Dan Liao, Jihua Zeng, Yuhong Yang

**Affiliations:** ^1^ Department of Psychiatry, Wuhan Wudong Hospital, Wuhan, China; ^2^ Department of Psychiatry, Affiliated Wuhan Mental Health Center, Tong ji Medical College of Huazhong University Science and Technology, Wuhan, China

**Keywords:** methamphetamine, paliperidone palmitate long-acting injection, electroacupuncture treatment, addiction, brain wave

## Abstract

**Objective:** To explore the clinical efficacy of paliperidone palmitate long-acting injection combined with electroacupuncture in the treatment of methamphetamine addicts.

**Methods:** This study focused on methamphetamine addicts who were admitted to our hospital from January 2020 to December 2020 as the main research object, with a total of 89 cases. The patients were divided into a control group of 45 cases and a study group of 44 cases according to the treatment method. The control group was treated with electroacupuncture, and the study group was treated with paliperidone palmitate long-acting injection on the basis of electroacupuncture in the control group. After 6 months of continuous treatment, the treatment effect of methamphetamine withdrawal symptom score before and after treatment was used; Hamilton Anxiety Scale score and Hamilton Depression Scale were used to compare the anxiety and depression situation of the two groups; the brain wave α and θ wave situation of the two groups were compared.

**Result:** The results showed that there was no significant difference in the scores of Ma withdrawal symptoms, Hamilton Anxiety and Hamilton Depression between the two groups before treatment (*p* < 0.05); after 3 and 6 months of treatment, the scores of Ma withdrawal symptoms, Hamilton Anxiety and Hamilton Depression in the study group were significantly lower than those in the control group and the difference was statistically significant (*p* < 0.05); 6 months after the completion of the treatment, the α wave amplitude and Fourier transformed α brain wave (FFT_α_) in the study group were significantly higher than those in the control group, and the difference was statistically significant (*p* < 0.05).

**Conclusion:** Paliperidone palmitate long-acting injection combined with electroacupuncture is better than electroacupuncture alone in the treatment of methamphetamine addicts, and can significantly improve anxiety, depression and brain waves, thereby preventing addicts from relapse.

## Introduction

Methamphetamine (METH) is a pure white crystal with a similar appearance to ice. It is an amphetamine type central nervous stimulant (Morgan et al., 2010). METH has a great influence on cerebral blood flow perfusion, glucose metabolism, some biochemical substances and even local brain structure, and it is highly addictive. METH drugs mainly include heroin, opium, etc. Although the physical symptoms after withdrawal are mild, mental withdrawal is more difficult, and most patients relapse due to mental withdrawal symptoms (Rezaei and Saadat, 2019; Dasgupta, 2020; Ghiabi, 2020). Paliperidone palmitate long-acting injection is an atypical anti-psychotic long-acting injection (Kim et al., 2012; Attard et al., 2014; Nikoli et al., 2016; Nikoli et al., 2017). Paliperidone palmitate can be hydrolyzed into paliperidone in the body, which inhibits mental excitement and related psychiatric diseases by antagonizing central dopamine D2 receptors and serotonin 2 receptors (Miller et al., 2019; Entescu, 2020). At present, paliperidone palmitate long-acting injection has been widely used in the treatment of schizophrenia and other diseases. Theoretically, paliperidone palmitate long-acting injection can prevent the relapse of METH addicts by regulating the nervous system, but there are not many clinical studies on this aspect. Paliperidone palmitate long-acting injection is a new type of anti-schizophrenia long-acting injection. It is currently commonly used in the treatment of acute and maintenance schizophrenia. Once a month, it is beneficial to improve the compliance and non-compliance of patients with medication. Affect the work and life of patients and return to society as soon as possible. After treatment, patients with methamphetamine addiction will experience withdrawal symptoms such as anxiety, irritability and restlessness. The relief of anxiety symptoms can prove to support the reduction of withdrawal symptoms, thereby reducing relapse. Methamphetamine addiction patients often have the same symptoms as patients with schizophrenia, so it could choose the same treatment drug paliperidone palmitate long-acting injection.

Therefore, this study takes methamphetamine addicts admitted to our hospital as the main research object, and intends to explore the clinical efficacy of paliperidone palmitate long-acting injection combined with electroacupuncture in the treatment of methamphetamine addicts.

## Materials and Methods

### Research Object

This study focused on methamphetamine addicts who were admitted to our hospital from January 2020 to December 2020 as the main research object, with a total of 89 cases. The patients were divided into a control group of 45 cases and a study group of 44 cases according to the treatment method. The control group was treated with electroacupuncture, and the study group was treated with paliperidone palmitate long-acting injection on the basis of electroacupuncture in the control group. This study complies with the “Declaration of Helsinki of the World Medical Association” and has been approved by the ethics committee of our hospital. All patients signed an informed consent form.

### Inclusion and Exclusion Criteria

Inclusion criteria 1) METH abuse for more than 6 months and addiction history for more than 6 months; 2) Aged over 18 years old and under 70 years old; 3) Able to quietly cooperate with the test and understand the meaning of each test; 4) Patients who have signed an informed consent form.

Exclusion criteria 1) Having a history of allergies to the drugs used in this experiment; 2) Having serious diseases of the heart, liver, lung, kidney and other organs; 3) Women during lactation or pregnancy. 4) Patients who cannot continue treatment due to external factors; 5) Patients with incomplete case data.

### Research Methods

The control group was treated with electroacupuncture, taking the bilateral Neiguan, Shenmen, Zusanli, Sanyinjiao, Huatuo Jiaji points on both sides of the patient, and inserting the needles with one hand, piercing 0.5–0.8 inches, and each needle was connected to low-frequency electronic pulse therapy instrument (model: G6805-2B; purchased from: Shanghai Huayi Co., Ltd.). Select the continuous pulse wave with a stimulation frequency of 2 Hz, and the stimulation intensity is subject to the maximum acceptable to the patient. The needle is retained for 20 min. Once every other day, the treatment was continued for 3 months for a total of 45 times.

**FIGURE 1:**
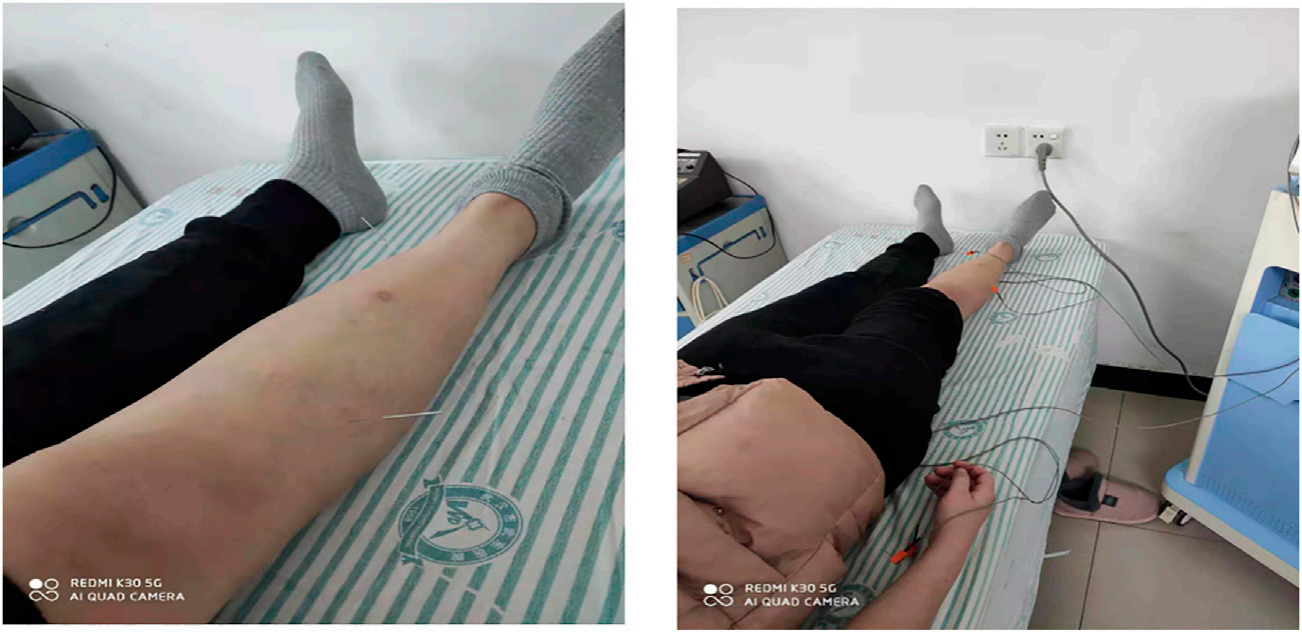
Electroacupuncture operation diagram.

On the basis of the electroacupuncture treatment in the control group, the study group was treated with paliperidone palmitate long-acting injection, and the long-acting injection of paliperidone palmitate (manufacturer: Janssen Pharmaceutica N.V.; drug standard code 86979438000138) 150 mg was injected on the first day, and 100 mg was injected again a week later, all at the deltoid muscle. After two injections, it was injected once a month. The injection dose was 100 mg. The injection site could be the deltoid muscle or the gluteal muscle. The treatment time was 3 months. See Figure 1 for details.

### Main Observation Indicators and Evaluation Criteria

In each group of patients, the changes in METH withdrawal symptoms were evaluated according to the METH withdrawal symptom scale before treatment, the 3 month after treatment, and the 6 month after treatment. Evaluate patients according to the 14 items of the withdrawal symptom score scale compiled by the China Institute of Drug Dependence of Peking University (Zhang, 1998), 1 point is for mild or occasional symptoms that do not require special treatment; 2 is for moderate symptoms; 3 is for severe symptoms that are affected by this disease most of the day.

The anxiety of patients in each group was evaluated according to Hamilton Anxiety Scale (HAMA) before treatment, 3 months after treatment and 6 months after treatment. Patients were evaluated according to the 14 items of HAMD compiled by Hamilton in 1959. Each item is scored from 0 to 4 points, among which 0 is asymptomatic; 1 is mildly symptomatic; 2 is moderately symptomatic; 3 is severely ill; 4 points for extremely severe symptoms. A total score of ≥ 29 points may be severe anxiety; ≥ 21 points, there must be obvious anxiety; ≥ 14 points, there must be anxiety; more than 7 points, there may be anxiety; if less than 7, there are no anxiety symptoms.

The depression of patients in each group was evaluated according to the Hamilton Depression Scale (HAMD) before treatment, 3 months after treatment, and 6 months after treatment. Patients were evaluated for depression according to the 17 items of HAMD compiled by Hamilton in 1960.0 is asymptomatic; 1 is mild; 2 is moderate; 3 is severe; 4 is very severe. If the total score is less than 7, it is normal; if the total score is 7–17, it is possible to have depression; if the total score is 17–24, it is certain to have depression; if the total score is more than 24, it is serious depression.

After 6 months of treatment, after all patients entered the treatment room and rested for 5 min, a multifunctional biofeedback instrument (model: spririt-10; purchased from: spirit company in the Netherlands) was used to collect brainwave signals for each group of patients. Biotrace software was used to analyze the signals to obtain a relatively stable average value of the physiological signals of each stage of each treatment.

### Statistical Analysis

In this study, SPSS 22.0 statistical software was used for data processing, and the measurement data were expressed as mean ± standard deviation (‾x ± s). Counting data is expressed in percentage (%). The comparison between the two groups that obey the normal distribution adopts the t-test; the comparison between the groups that does not obey the normal distribution adopts the non-parametric test. Chi-square test is used for counting data. *p < 0.05* indicates that the difference is statistically significant.

## Results

### General Information

A total of 89 cases of METH addicts were included in this study, and the patients were divided into a control group of 45 cases and a study group of 44 cases according to the treatment method. Among them, there were 22 males in the control group and 23 females; the ages were 18–58 years old, with an average of (37.21 ± 10.25); METH abuse time was 6–58 months, and the average abuse time was (31.25 ± 11.33) months. There were 21 males and 23 females in the study group; the ages were 19–54 years old, with an average of (35.691 ± 11.18); METH abuse time was 6–66 months, and the average abuse time was (32.78 ± 10.97) months. There was no statistically significant difference in general information such as age and gender between the two groups (*p* > 0.05).

### Comparison of the Methamphetamine Withdrawal Symptom Score Scale Between the Two Groups

There was no significant difference in METH withdrawal symptom scores between the study group and the control group before treatment (*p > 0.05*), after 3 months of treatment (12.07 ± 2.09 vs. 19.00 ± 3.07) and after 6 months of treatment (8.39 ± 1.07 vs. 15.36 ±2.39). The scores of METH withdrawal symptoms in the study group were significantly lower than those in the control group, and the difference was statistically significant (*p* < 0.05), see Table 1.

**TABLE 1:** The comparison about MA withdrawal symptom scores between the two patient groups (‾x ± s).

Group	MA withdrawal symptom score before treatment	MA withdrawal symptom score after 3 months of treatment	MA withdrawal symptom score after 6 months of treatment
The control group (*n* = 45)	25.36 ± 2.46	19.00 ± 3.07	15.36 ± 2.39
The study group (*n* = 44)	26.07 ± 2.28	12.07 ± 2.09	8.39 ± 1.07
T score	1.411	12.421	17.687
P score	0.167	0.000	0.000

### Comparison of Anxiety Between the Two Groups

There was no significant difference in Hamilton anxiety score between the study group and the control group before treatment (*p* > 0.05), after 3 months of treatment (19.04 ± 3.86 vs. 24.01 ± 2.99) and after 6 months of treatment (10.36 ± 1.90 vs. 18.31 ± 2.47). The Hamilton anxiety scores of the study group were significantly lower than those of the control group, and the difference was statistically significant (*p* < 0.05), see Table 2.

**TABLE 2:** The comparison about the Hamilton anxiety scores between the two patient groups (‾x ± s).

Group	HAMA before treatment	HAMA after 3 months of treatment	HAMA after 6 months of treatment
The control group (*n* = 45)	28.98 ± 4.70	24.01 ± 2.99	18.31 ± 2.47
The study group (*n* = 44)	30.01 ± 3.00	19.04 ± 3.86	10.36 ± 1.90
T score	1.229	6.800	16.992
P score	0.222	0.000	0.000

### Comparison of the Degree of Depression in the Two Groups

There was no statistically significant difference in Hamilton depression score between the study group and the control group before treatment (*p* > 0.05), after 3 months of treatment (15.36 ± 2.07 vs. 19.39 ± 2.27) and 6 months after treatment (11.01 ± 1.70 vs. 15.69 ± 2.95). The Hamilton depression scores in the study group were significantly lower than those in the control group, and the difference was statistically significant (*p* < 0.05), see Table 3.

**TABLE 3:** The comparison about the Hamilton depression scores between the two patient groups (‾x ± s).

Group	HAMD before treatment	HAMD after 3 months of treatment	HAMD after 6 months of treatment
The control group (*n* = 45)	25.69 ± 2.36	19.39 ± 2.27	15.69 ± 2.95
The study group (*n* = 44)	26.33 ± 3.17	15.36 ± 2.07	11.01 ± 1.70
T score	1.082	8.746	9.142
P score	0.282	0.000	0.000

### Comparison of EEG Indicators Between the Two Groups

After 6 months of treatment, the EEG indicators of the two groups of patients were tested. The α wave amplitude and Fourier transformed α EEG (FFT_α_) of the study group were significantly higher than those of the control group (12.35 ± 2.11 vs. 8.36 ± 1.97), and the difference was statistically significant (*p* < 0.05); the θ wave amplitude in the study group (12.39 ± 4.09 vs. 22.58 ± 5.32) and Fourier transformed θ brainwave (FFT^θ^) (8.65 ± 1.08 vs. 19.36 ± 3.68) were significantly lower than those of the control group, the difference was statistically significant (*p* < 0.05), see Table 4.

**TABLE 4:** The comparison about the EEG indicators between the two patient groups (‾x ± s).

Group	[Α]	[FFT_α_]	[θ]	[FFT_θ_]
The control group (*n* = 45)	12.36 ± 4.78	8.36 ± 1.97	22.58 ± 5.32	19.36 ± 3.68
The study group (*n* = 44)	19.21 ± 5.54	12.35 ± 2.11	12.39 ± 4.09	8.65 ± 1.08
T score	6.250	9.224	10.141	18.659
P score	0.000	0.000	0.000	0.000

## Discussion

After withdrawal from the use of MA, patients who abuse METH often suffer from uninteresting, unexplainable depression, anxiety, unwillingness to deal with people, and slow response. When the patient relapses, there will be a sense of excitement and pleasure, and will have great dependence on this. In order to judge the patient’s METH withdrawal status, the METH withdrawal symptom score can be used from the 14 aspects of appetite, fatigue, drowsiness, medication desire, drowsiness, interest in communication, concentration, anxiety, depression, labor or work, study, self-control, nausea, headache and suicidal thoughts to evaluate the patient’s withdrawal status. This study found that the scores of METH withdrawal symptoms in the study group were significantly lower than those in the control group after 3 months of treatment and 6 months of treatment. It shows that paliperidone palmitate long-acting injection can effectively help patients with METH abuse to withdraw from addiction.

METH has a sympathomimetic mechanism. It enters the brain and exerts a strong central excitatory effect. Whenever METH is used, it will stimulate the brains of long-term METH abusers to produce excitement and pleasure (Alexander, 2019; Demirci et al., 2020; Hill et al., 2020; Willis and Painter, 2020). Discontinuing METH for a period of time will make the patient feel depressed, at the same time there will be insomnia, pessimism, world weariness, upset and night sweats. Anxiety, depression, etc. will appear after a period of time. In this study, the Hamilton Depression and Anxiety Score Scale was used to detect anxiety and depression of the two groups of patients. It was found that the Hamilton depression scores of the two groups of patients decreased significantly over time. It shows that with the progress of treatment, the patient’s anxiety and depression can be relieved to a certain extent, so that the patient's desire for the pleasure of relapse of METH has a certain degree of reduction. At the same time, the Hamilton depression score of the study group was significantly lower than that of the control group, indicating that the use of paliperidone palmitate long-acting injection can further reduce the patient’s anxiety and depression, further reduce the patient’s dependence on METH smoking, and can effectively prevent the patient from relapse. Past use of electroacupuncture has better improvement in METH withdrawal symptoms and emotional anxiety, which is consistent with the conclusions drawn in this study (Ting-Ting et al., 2009; Cui et al., 2013; Ma et al., 2015).

Brain waves will produce different amplitudes with different mental states. Among them, the α wave (Alpha wave) is 8–12 Hz, which is most obvious when people are quiet, relaxed, and not excited. The θ wave (Theta wave) is 4–8 Hz. This brain wave will increase significantly when a person is depressed. The abuse of METH will cause damage to the brain structure and make normal brain waves abnormal (Lee and Pau, 2002; Gekht et al., 2003; Zhang et al., 2011). This study showed that the α wave amplitude and Fourier transformed α brain wave (FFT_α_) of the study group were significantly higher than those of the control group, and the θ wave amplitude and Fourier transformed θ brain wave (FFT_θ_) amplitude were significantly lower than those of the control group. It shows that the patients in the study group are calmer after METH withdrawal, and have less negative emotions such as depression. This shows that the patients in the study group are less dependent on the excitement of METH after treatment, their mentality is more peaceful, and the probability of relapse is lower.

This research still has the following shortcomings. First of all, this study is not a randomized controlled experiment, and there is no blinding method, so there is still a certain risk of bias. Secondly, this study is a single-center clinical study, and subsequent multi-center clinical studies are still needed for further discussion. Finally, the sample size included in this study is relatively small, and it is still necessary to increase the sample size for further research.

In conclusion, paliperidone palmitate long-acting injection combined with electroacupuncture is better than electroacupuncture alone in the treatment of methamphetamine addicts, and can significantly improve anxiety, depression and brain waves, thereby preventing addicts from relapse.

## Data Availability

The original contributions presented in the study are included in the article/Supplementary Material, further inquiries can be directed to the corresponding authors.
